# Proportion of stillbirth and associated factors among women who deliver at public hospitals in Bahir Dar city, north-West Ethiopia

**DOI:** 10.1186/s12905-024-02920-8

**Published:** 2024-02-16

**Authors:** Bantayehu Nega Arega, Lakachew Asrade Feleke, Hiwotemariam Alemu Tilahun, Dawud Muhammed Ahmed, Fekadie Getachew Hailu

**Affiliations:** Department of obstetrics and gynecology, college of medicine and health sciences, Bahir Dar University, Bahir Dar, Ethiopia

**Keywords:** Still birth, Proportion, Associated factors

## Abstract

**Introduction:**

The annual global burden of stillbirths is estimated to be 3.2 million, of which 98% occur in low and middle-income countries (LMICs). In the Amhara region of Ethiopia, the prevalence of stillbirth outcomes was 85 per 1000. Ethiopia is experiencing an increase in the number of health professionals attending deliveries, however, stillbirth rates are not decreasing as anticipated. However, there are limited numbers of studies done related to the proportion of stillbirths and associated factors in the study area. This study aimed to assess the proportion of stillbirths and associated factors among women who attended deliveries at Tibebe Ghion Specialized Hospital and Felege Hiwot Comprehensive Specialized Hospital.

**Methods:**

An institutional-based cross-sectional study was conducted on 366 women who delivered at two referral hospitals in Bahir Dar from April 1, 2020, to August 30, 2020. Study participants were selected using systematic random sampling techniques. A checklist and structured questionnaire were used to retrieve information from the clients and their attendants. The collected data were cleaned, coded, and entered into Epi-data version 3.1 and then exported into SPSS 23 for analysis. Bivariate and multivariable logistic regression analysis was computed to identify statistically significant associated factors with a *P* value < 0.05. The results were presented in tables and charts.

**Result:**

The proportion of stillbirths was 3.8% in this study area. This study showed that level of education, who completed primary school (AOR = 0.12; 95% CI (0.01, 0.98)), not using partograph (AOR = 3.77, 95%; CI (1.02; 13.93)), and obstetric complication (AOR = 6.7; 95% CI (1.54, 29.79) were the major factors affecting the stillbirth.

**Conclusion:**

Our study found that stillbirth rate remains a major public health problem. Illiteracy, not using a partograph, and having obstetric complications were major associated factors for stillbirth. The risk factors identified in this study can be prevented and managed by providing appropriate care during preconception, antepartum, and intrapartum periods.

## Introduction

Stillbirth is defined as a baby born with no signs of life, weighing more than 1000 g, or with more than 28 completed weeks of gestation. Fetal death can be intrapartum or antepartum [1]. The WHO update estimates that 2.6 million stillbirths occurred in 2015 [2, 3]. A global stillbirth rate of 13.9 stillbirths per 1000 total births was estimated in 2021, with an estimated 1.9 million babies stillborn at 28 weeks of pregnancy or later [4]. According to the 2016 Ethiopian demographic and health survey, the national stillbirth rate was 11.8 per 1000 pregnancies [5], while the Amhara region, where the study will be done, had a stillbirth rate of 85 per 1000 pregnancies [6].

Stillbirth is correlated with profound adverse outcomes, including psychological and social expenses incurred by women and their families, the community, and the government. These women are afflicted with anxiety, persistent depression, post-traumatic stress disorder, and stigmatization [7–10]. Stillbirth is a major adverse birth outcome that affects both developing and developed countries. Stillbirth due to intra-partum loss is higher in developing countries than in developed countries where it is 59 and 10%, respectively. It has been reported that low- and middle-income countries account for 99% of these deaths [11].

Decreasing the global burden of stillbirth mainly focuses on strategic interventions, and to enable these strategic interventions to minimize the stillbirth rate, identification of risk factors for stillbirth is needed. Previous studies have identified several factors linked to the occurrence of stillbirth. These factors include lack of prenatal care, age at first birth, birth order number, and the preceding birth interval, drinking alcohol during pregnancy, antepartum hemorrhage, premature rupture of membrane, meconium-stained amniotic fluid, induction of labor, labor not followed by partograph, previous history of stillbirth, and a birth weight less than 2500 g [12–18]. However, these factors may vary across countries and time trends depending on the quality and accessibility of care in the health facility, and estimates for stillbirth determinants are impeded by various classification systems because of the unavailability of reliable data. As a result, the stillbirth rate has decreased, but only by very small amount, even though many developing nations, including Ethiopia, have been adopting many efficient programs to promote maternal and child health and build the abilities of health professionals [19].

By 2030, the Every Newborn Action Plan goal of 12 stillbirths per 1000 live births will not be achieved in developing nations if the current rates of decline continue. To achieve every newborn action plan goal of stillbirth reduction, more focus will be needed on the risk factors and treating the causes of stillbirth [15]. The Ethiopian government has been putting in place a variety of successful programs to enhance mother and child health conditions and strengthen the capacity of health workers to improve the quality of service during pregnancy, such as prenatal and delivery care [19]. Although stillbirth and newborn mortality have shown less progress in Ethiopia, the outcome during pregnancy and delivery periods remains a critical issue in achieving the stated target in the Sustainable Development Goals (SDGs) [20, 21]. However, the information shown in the study area is limited. As a result, the findings of this study will assist policymakers, program planners, implementers of governmental and non-governmental organizations, and providers and practitioners of maternal health services in offering evidence-based interventions that will help reduce the number of stillbirths in the hospital and the surrounding area.

## Methods

### Study area and period

An institutional-based cross-sectional study was conducted from April 1, 2020, to August 30, 2020, GC, at FHCSH and TGSH in Bahir Dar Amhara Regional State, North-West, Ethiopia. Bahir Dar is the capital city of Amhara National Regional State, located 565 km Northwest of Addis Ababa with an altitude of 1799 m above sea level a warm and temperate climate with an estimated population of 168,899 as per the 2018 world population review.

### Study design

An institutional-based cross-sectional study.

### Population

#### Source population

Any women who visited FHCSH and TGSH for delivery services from April 1, 2020, to August 30, 2020, G.C.

#### Study population

Women who gave birth at TGSH and FHCSH in the study period were included.

### Inclusion criteria

Women who gave birth at FHCSH and TGSH who were randomly selected during the study period are included in the study.

### Exclusion criteria

Women who died after delivery and before data collection were excluded.

### Sample size estimation and sampling technique

The single population proportional formula was used to calculate the sample size. The total sample size was calculated using the following assumption to come up with the final sample size.

Confidence level = 97%.

The margin of error (precision) = 3%.

Proportion of stillbirth (p) = 8.5% [6].

$$n=\frac{z^2\ p\left(1-p\right)}{d^2}$$

Where n = sample size, *p* = 0.085, d = 0.03(3% error of margin), z = 1.96 (standard normal probability for 97% CI) with a 10% non-respondent included, the sample size was 366.

A systematic random sampling technique was used to select study participants from the register within the referral hospitals, the sampling fraction was 1 /9 and every 9th was involved. A lottery system was used to determine the first mother from the delivery register.

The share of each hospital was determined based on the number of clients from the previous 6 months’ report. The calculated share was as follows: FHCSH = 248, TGSH = 118.

Data were collected by first-year residents and interns and supervised by a senior resident.

## Study variables

Dependent variable Stillbirth (yes/No).

### Independent variables

Socio demographic factors(age, marital status, educational status of the mother, residence, Socio-economic status, maternal occupation, religion),obstetric factors (parity, gravidity, gestational age, ANC, pregnancy-related complications, type of gestation, mode of delivery, pregnancy status, previous history of abortion, partograph use, labor abnormality, previous history stillbirth), and medical problems.

### Operational definition

Bad obstetric history: Mothers who had a history of LBW, preterm birth, stillbirth, perinatal death, or abortion [22].

Obstetric complication: pregnant women who had pregnancy induced hypertension(preeclampsia or eclampsia), premature rupture of membrane, antepartum hemorrhage or gestational diabetes mellitus.

Low birth weight: Birth weight less than 2.5 kg [1].

Preterm birth: Delivery that occurs before 37 weeks of gestation [1, 22].

Stillbirth: It is a baby born with no signs of life at or after 28 weeks gestation [22].

### Data collection

A structured interviewer-administered questionnaire was adopted from different literature [18, 22–25]. Data were collected from mothers using structured checklists and questionnaires. First, the questionnaire was prepared in English and translated to the local language, Amharic, and translated back to English to observe its consistency. Finally, the questionnaire was pre-tested on 5% of mothers at Debre Markos referral hospital before the actual data collection; correction and modification were done based on the gap identified during the pre-test interview. The check list on the questioner was checked by data collectors & supervisors daily for completeness.

### Data processing and analysis

Data were entered into Epi-data version 3.1 and then transported to SPSS 23 software packages for analysis. Descriptive statistics such as mean and percentage were determined. To identify associated factors of stillbirth, binary logistic regression was conducted, and variables with a *p* value less than 0.2 were selected for multivariable logistic regression. The *p*-value of 0.05and the 95% confidence interval (CI) were chosen as the level of significance. The results were described using tables, pie charts, and other graphs.

### Data quality control

Before data collection, the checklist was tested to check the consistency of the format and the ability of the data collector’s performance. The checklist was modified based on the pretest results. One day of training and orientation was given to data collectors on how to carry out data collection and quality control.

## Results

### Socio-demographic and socio-economic characteristics of respondents

In this study, a total of 366 participants were involved. Two hundred ninety-five (80.6. %) of the study participants were in the age group of 20–34 years and 325 (88.8%) were Orthodox followers. Most of the respondents (*n* = 202; 55.2%) were Urban, and 346(94.5%) were married. One hundred seventy-seven (48.4%) of the study participants were housewives, and Two hundred five (56%) had a monthly income of less than 5000 Ethiopian birrs. Regarding educational status, the majority, 106(29%) were illiterate and 97(26.5%) had completed primary school. Ethnically, the majority of 364(99.5%) were from the Amhara region (Table 1).

**Table 1:** Socio-demographic characteristics of respondents assessed on stillbirth in Tibebe Ghion and Felege Hiwot Specialized Hospital, Bahir Dar, Ethiopia, 2020

Characteristics	Variable	Total Respondents [((Both Women with stillbirth +alive birth)]	
		Frequency	Percent (%)
Age(yrs)	< 20	16	4.4
	20–34	294	80.3
	> 34	56	15.3
	Total	366	100
Residence	Urban	202	55.2
	Rural	164	44.8
	Total	366	100
Marital status	Single	12	3.3
	Married	346	94.5
	Divorced	8	2.2
Religion	Orthodox	325	88.8
	Muslim	37	10.1
	protestant	4	1.1
Monthly family income	< 5000	205	56.0
	5000–10,000	116	31.7
	> 10,000	45	12.3
Occupation	Housewife	177	48.4
	Government employ	52	14.2
	Farmer	40	10.9
	Others*	97	26.6

Others* = Student, private employ, merchant

### Gynecological and obstetrical related characteristics of respondents

Among the total respondents, 205 (56%) were primi para and 109(29.8%) were multipara. Of the total respondents, 358 (97.8%) had ANC follow-ups, and 220(60.1%) were at the health center, 285(77.9%)mothers have not faced any obstetric complications during the current pregnancy. According to this study, Most of the mothers (192 (52.5%)) had attended ANC less than four times, and 166(45.4%) had attended ANC four times and more. Most of the mothers (181; 49.5%) started ANC in the first trimester of the current pregnancy.

**Fig. 1:**
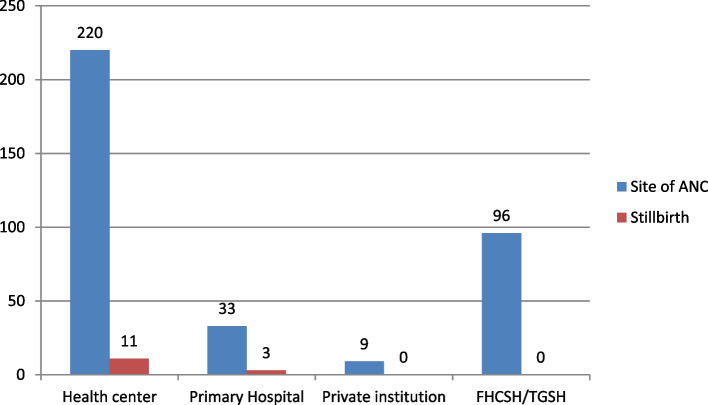
A bar graph that shows the site of ANC follow-ups in comparison with the stillbirth rate for women who attended deliveries at two public hospitals in Bahir Dar, Ethiopia, 2020

Regarding a history of poor obstetric outcomes, 53 (14.5%) of participants had faced a history of perinatal loss in their previous pregnancies. One hundred two (27.9%) mothers encountered obstetric complications during recent pregnancy, comprising PIH 66(18%) followed by APH 22(6%). Among all deliveries, 44(12%) experienced labor abnormalities (dystocia). Two hundred thirty-three (63.7%) of current deliveries were vaginal deliveries, followed by cesarean sections (CS) 129(35.2%). Among the respondents, 240(65.6%) were followed with a partograph. Twelve (3.3%) mothers had medical illnesses during the current pregnancy, including cardiac diseases 5(1.4%). The findings of this study showed that the prevalence of stillbirth was 3.8% **(**Table 2**)**(Fig. 1).

**Table 2:** Gynecological and obstetric, and newborn characteristics of respondents in Tibebe Ghion and Felege Hiwot Specialized Hospital, Bahir Dar, Ethiopia, 2020

Characteristics	Variable	Total Respondents [((Both Women with stillbirth +alive birth)]	
		Frequency	Percent (%)
Gravidity	1	172	47.0
	2–4	136	37.2
	> 5	58	15.8
	Total	366	100
Parity	1	205	56
	2–4	109	29.8
	> 5	52	14.2
Alive children	0	20	5.5
	1–4	302	82.5
	> 5	44	12.0
Gestational age of current pregnancy	28–37 weeks	52	14.2
	37–42 weeks	256	69.9
	> 42 weeks	24	6.6
	unknown	34	9.3
	Yes	358	97.8
	No	8	2.2
Number of visits to the ANC	Less than 4	192	52.5
	4 and above	166	45.4
Start of first ANC	First trimester	181	49.5
	Second trimester	142	38.8
	Third trimester	29	7.9
		6	1.6
Problem detected during ANC follow-up,	Yes	73	19.9
	No	285	77.9
Mode of delivery	CS	129	35.2
	Operative Vaginal	4	1.1
	Vaginal	233	63.7
Labor abnormality	Yes	44	12.0
	No	291	79.5
Partograph use	Yes	240	65.6
	No	93	25.4
Type of medical disease	HIV	2	o.5
	Cardiac disease	5	1.4
	Hypertension	2	0.5
	DM	3	0.8
Obstetric complication	yes	102	27.9
	No	264	72.1
Type of obstetric complication	PE/E	66	18.0
	APH	22	6.0
	PROM	10	2.7
	GDM	4	1.1
Birth outcome	Alive	352	96.2
	Stillbirth	14	3.8

### Factors associated with stillbirth

The entire variable was analyzed with binary logistic regression, and of all the variables, the number of pregnancies, number of deliveries, history of previous abortions, number of alive children, level of education, photograph use, type of religion, and pregnancy-related obstetric complications had a *p*-value of < 0.2. In multivariable logistic regression analysis, the number of deliveries, number of pregnancies, history of previous abortions, religion, and number of alivechildren had no association with stillbirth. But the level of education, partograph use, and obstetric complications had shown a significant association with stillbirth with a *p*-value < 0.05.Mothers who were not followed with partograph were almost 4 times more likely to give stillbirth compared to those followed with partograph [AOR = 3.77, 95% CI (1.02, 13.93)]. Newborns delivered from those mothers who had completed primary school and above were 88% less likely to be stillborn as compared to those delivered from illiterate mothers [AOR = 0.12, 95% CI (0.01, 0.98)].This study shows newborns who were born to mothers with obstetric complications are almost seven times more likely to be stillborns compared to those who were born from mothers with no obstetric complications (AOR = 6.72; 95% CI (1.54, 29.79) (Table 3).

**Table 3:** Factors associated with stillbirth among deliveries at Tibebe Ghion and Felege Hiwot Specialized Hospital, Bahir Dar, Ethiopia, 2020

	Stillbirth				
Variable	YES	NO	COR (CI-95%)	AOR (CI-95%)	*p*-value
Gravidity					
1	5(35.7%)	167(47.4%)	1	1	
1–4	2(14.3%)	134(38.1%)	0.49(0.09,2.61)	0.22 (0.03,1.53)	0.12
> 5	7(50%)	51(14.5%)	4.58(1.39,15.06)	3.38(0.23,48.32)	0.36
Parity					
Less than 4	8(57.1%)	306(87%)	1	1	
5 and above	6(42.9%)	46(13%)	4.98(1.65,15.03)	0.64(0.02,10.74)	0.63
History of abortion					
Yes	5(35.7%)	31(8.8%)	1	1	
No	9 (64.3%)	321(91.2%)	0.17(0.05,0.55)	0.22(0.04,1.23)	0.86
No alive child					
Less than 4	11(78.6%)	311(88.4%)	1	1	
5 and above	3(21.4%)	41(11.6%)	2.06(0.55,7.72)	0.23(0.02,2.55)	0.23
Obstetric complication					
NO	5(35.7%)	259(73.6%)	1	1	
YES	9(64.3%)	93(26.4%)	5.02(1.63,15.34)	6.72(1.54,29.79)	0.01
Education					
Illiterate	7(50%)	99(28.1%)	1	1	
Can read & write	4(28.6%)	62(17.6%)	0.91(0.25,3.24)	0.72(0.14,3.52)	0.68
Primary school	2(14.3%)	95(27.0%)	0.29(0.06,1.47)	0.12(0.01,0.98)	0.04
High school & above	1(7.1%)	96(27.3%)	0.30(0.03,2.52)	0.17(0.01,2.40)	0.16
Partograph use					
NO	7(50%)	86(24.4%)	2.70(0.92,7.95)	3.77(1.02,13.93)	0.04
YES	7(50%)	233(66.2%)	1	1	
Religion					
Orthodox	9(64.3%)	315(89.5%)	1	1	
Muslim	4(28.6%)	33(9.4%)	3.8(1.13,12.85)	5.59(1.13,27.58)	0.06
Others**	1(7.1%)	4(1.1%)			

Others** = Protestant, Catholic

## Discussion

In this study, the proportion and associated factors of stillbirth were assessed in two public hospitals in Bahir Dar, Amhara region and the result of the study revealed that the proportion of stillbirths was 3.8%. This finding was similar to a studyconducted in Aksum General Hospital, Tigray region, which shows a prevalence of 3.68% [22], in Tanzania, which was 3.5% [26], and in Nigeria at 4.8% [27]. Whereas lower than the studies conducted at the Buea Regional Hospital Fako Division south-west region, Cameroon, in Jimma University specialized hospital, Ethiopia and Amhara region using the Ethiopian Mini Demographic and Health Survey (EMDHS) which shows 26,8, and 8.5%, respectively. The variations between these findings may be due to the socioeconomic variations of the study subjects, of whom most them were urban residents which may result in improved birth outcome [28].

This study showed that level of education, partograph use, and obstetric complications were significantly associated with the likelihood of having a stillbirth. Mothers with obstetric complications (like pregnancy-induced hypertension, Antepartum hemorrhage, premature rupture of fetal membranes, and GDM) in recent pregnancies were found to have a higher chance of experiencing stillbirth than those without obstetric complications. This finding was consistent with the study done in Nepal [29], India [30], Latvia [31], two sites each in Africa (Zambia and Kenya) [15], in ESIC MC and PGIMSR, Rajajinagar, Bangalore, Karnataka, Calabar, Nigeria’s University of Calabar Teaching Hospital (UCTH) [27], tertiary hospital in sub-Saharan Africa [32, 33], the Kilimanjaro Christian Medical Centre birth registry, Tanzania [26], Tigray [25], and Felege Hiwot Comprehensive Specialized Referral Hospital [18]. This may be explained by the fact that the complications that have occurred during pregnancy have affected the well-being of the fetus in the uterus and may lead to preterm termination of pregnancy.

Study participants who were not followed with partograph were more likely to have a stillbirth than mothers who were followed with partograph. This finding had similarities with studies done in Nepal [24], Aksum [22], and Felege Hiwot comprehensive specialized referral hospital [18].Those mothers who were not followed with partograph may have a delay in the detection of labor abnormalities, which could also result in delayed intervention.

Furthermore, newborns delivered by illiterate mothers were more likely to be stillborn than those who had completed primary school. This is consistent with the study done in Dhaka Bangladesh [13], Nepal [29], and the Amhara region using the Ethiopian Mini Demographic and Health Survey [5] and Felege Hiwot Comprehensive Specialized Referral Hospital [22].This may be explained in terms of the fact that illiterate mothers lack awareness of problems; lack early visits to health institutions, and may not understand given advice easily.

## Conclusion

Our study found that stillbirth rate remains a major public health problem, and it is far below to achieve Every Newborn Action Plan target by 2030. In this study, level of education, partograph use, and obstetric complications are the major factors for stillbirth. The risk factors identified in this study can be prevented and managed by providing appropriate care during preconception, antepartum and intrapartum periods. Detailed assessment and frequent follow-up for the mothers who had pregnancy-related complications, and a regular teaching program at the ANC to create awareness. The other intervention for every laboring mother is to use partograph to decrease preventable stillbirths.

## Data Availability

The data used for this study are available from the corresponding author and can be accessed with request.

## Abbreviations

ANC: Antenatal Care
AOR: Adjusted Odds Ratio
APH: Antepartum Hemorrhage
C/S: Cesarean Section
COR: Crude Odds Ratio
GDM: Gestational Diabetes Mellitus
FHCSH: Felege Hiwot Compressive Specialized Hospital
HIV: Human Immunodeficiency Virus
LBW: Low Birth Weight
PROM: Premature Rupture Of Membrane
SBR: Still Birth Rate
SVD: Spontaneous Vaginal Delivery
TGSH: Tibebe Ghion Specialized Hospital
WHO: World Health Organization
